# Robotic Total Mesorectal Excision for Low Rectal Cancer: A Narrative Review and Description of the Technique

**DOI:** 10.3390/jcm12144859

**Published:** 2023-07-24

**Authors:** Giampaolo Formisano, Luca Ferraro, Adelona Salaj, Simona Giuratrabocchetta, Gaetano Piccolo, Giulia Di Raimondo, Paolo Pietro Bianchi

**Affiliations:** 1 Department of Surgery, Asst Santi Paolo e Carlo, Dipartimento di Scienze della Salute, University of Milan, 20122 Milan, Italy; 2 Department of General Surgery, Asst Santi Paolo e Carlo, 20142 Milan, Italy

**Keywords:** robotic anterior resection, rectal cancer, total mesorectal excision, long-term outcomes

## Abstract

Robotic surgery may offer significant advantages for treating extraperitoneal rectal cancer. Although laparoscopy has been shown to be safe and effective, laparoscopic total mesorectal excision (TME) remains technically challenging and is still performed in selected centers. Robotic anterior resection (RAR) may overcome the drawback of conventional laparoscopy, providing high-quality surgery with favorable oncological outcomes. Moreover, recent data show how RAR offers clinical and oncological benefits when affording difficult TMEs, such as low and advanced rectal tumors, in terms of complication rate, specimen quality, recurrence rate, and survival. This series aims to review the most recent and relevant literature, reporting mid- and long-term oncological outcomes and focusing on minimally invasive RAR for low rectal cancer.

## 1. Introduction

The introduction of the embryologically based concept of total mesorectal excision (TME) by Heald more than 30 years ago has led to dramatic improvements in the local recurrence rates of patients with rectal cancer [1,2].

Despite continuous advances in multimodal treatment with refinements in imaging modalities and neoadjuvant therapy protocols (i.e., total neoadjuvant therapy), surgery still plays a significant role in being the strongest predictor of local recurrence (LR), disease-free survival (DFS), and overall survival (OS) [3].

Laparoscopic surgery has gained popularity during the last decades in treating rectal cancer. Three randomized controlled laparoscopic versus open rectal resection trials have demonstrated similar rates of circumferential resection margin (CRM) involvement, LR, DFS, and OS [4,5,6,7]. Nevertheless, the Australian Laparoscopic Cancer of the Rectum (ALACART) and the Effect of Laparoscopic-Assisted Resection vs. Open Resection of Stage II or III Rectal Cancer on Pathologic Outcomes (ACOSOG) trials have questioned the non-inferiority of laparoscopy when compared to open surgery in rectal cancer treatment [8,9].

The technical limitations of straight laparoscopic instruments in deep narrow spaces such as the pelvis, make laparoscopic TME a challenging procedure with a steep learning curve and a consistent conversion rate to open surgery. Therefore, efforts towards the optimization of minimally invasive surgical treatment of rectal cancer are needed. Robotic surgery may offer significant advantages thanks to its technological features. The wristed instruments may allow correct triangulation, traction, and counter traction even deep in the pelvis for a finer dissection and optimal tissue handling with a stable operative field [10,11]. These technical advantages have led other specialties to consider robotic surgery the mainstream approach for oncological pelvic surgery.

Several meta-analyses have highlighted the potential benefits of robotic surgery but have been criticized because of the low quality of the included studies. The obotic-Assisted vs. Conventional Laparoscopic Surgery on Risk of Conversion to Open Laparotomy Among Patients Undergoing Resection for Rectal Cancer (ROLARR) trial has recently failed to demonstrate a reduction in the conversion rate (primary endpoint) of robotic versus laparoscopic anterior resection for cancer but showed potential benefits in most challenging patient subgroups (male sex, high BMI, and low-lying tumors) [12]. On the other hand, the Chinese multi-center Robotic versus laparoscopic surgery for middle and low rectal cancer (REAL) trial has recently shown advantages in terms of the quality of oncological resection (CRM positivity), with better short-term and pathological outcomes of robotic surgery when compared to its laparoscopic counterpart [13]. Indeed, long-term outcomes are still pending for both trials.

The current paper aims to review the most recent and relevant literature comparing the oncological results of open, laparoscopic, and robotic TME.

## 2. Surgical Technique

**Figure 1:**
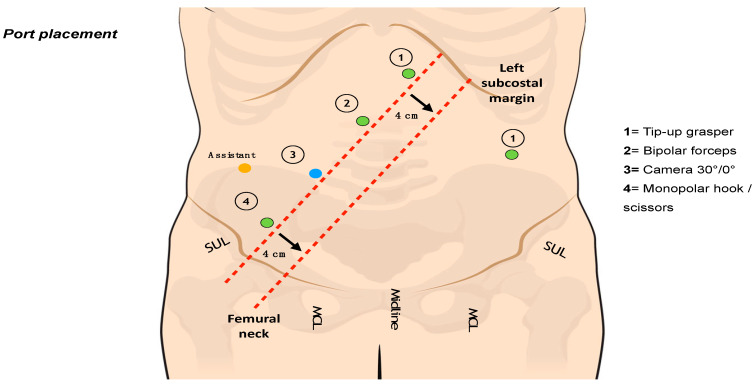
Trocar layout.

**Figure 2:**
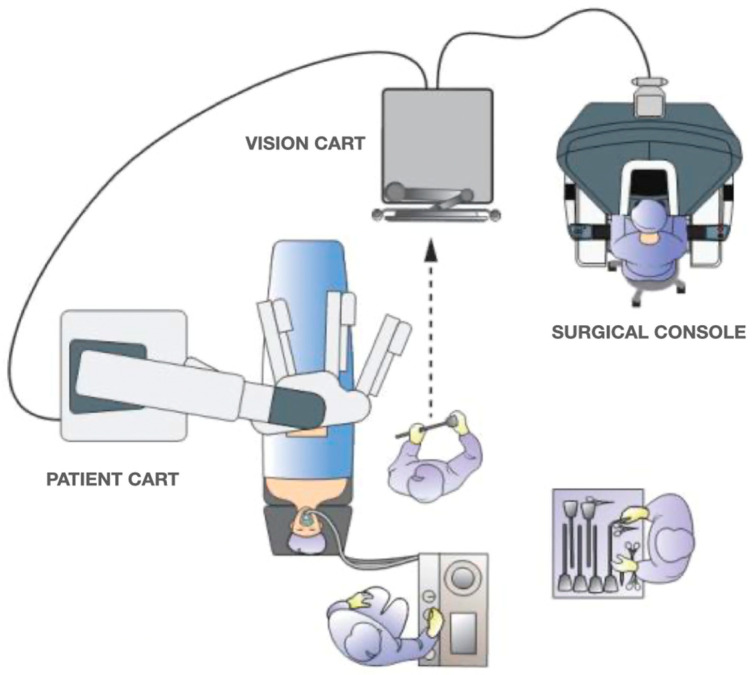
Operative room setup.

A Verres needle is inserted into the left hypochondrium (Palmer’s point) to induce 12 mmHg pneumoperitoneum. A 12 mm optical port is inserted into the right flank. Four 8 mm robotic trocars are then inserted along a straight line parallel to and about 4 cm cranial to the costofemoral line; a 6–8 cm distance between each port is maintained. An additional 8 mm robotic port is placed in the left flank and will be used for the TME. A 5 mm laparoscopic port may be introduced into the right hypochondrium to optimize the assistant’s tractions if required. The trocar layout is shown in Figure 1. The cart is deployed for docking from the patient’s left side. The operative room setup is shown in Figure 2.

The sigmoid colon is lifted with the robotic grasper in robotic arm number one (R1). The peritoneum is then incised at the level of the sacral promontory to access the avascular presacral mesorectal plane, where the hypogastric nerves are identified and preserved, performing a medial to lateral dissection up to identify the left ureter and gonadal vessels. The superior rectal artery is identified as a landmark, and the dissection continues bottom-to-up.

The inferior mesenteric artery’s (IMA) origin is thus identified with the surrounding lymphatic tissue, dissected at its origin, and divided with Hem-o-lok^®^ clips. The medial-to-lateral dissection is performed underneath the inferior mesenteric vein (IMV), and the Toldt–Gerota plane is identified as a landmark. The IMV is dissected, isolated, and transected using Hem-o-lok^®^ clips (Teleflex, Wayne, PA, USA). The dissection continues downward medial-to-lateral, with the R1-grasper lifting the descending-sigmoid mesocolon and the assistant surgeon providing counter traction on the Gerota’s fascia. Coloparietal detachment is then completed along the white line of Toldt. 

A splenic flexure takedown is performed with a one-inch one-inch bottom-up approach. The Toldt–Gerota plane previously developed and the IMV are identified. The transverse colon is lifted with an R1-grasper, and the lesser sac is opened through the incision of the transverse mesocolic root at the level of the anterior pancreatic border, gaining access to the lesser sac (one-inch one-inch bottom-up approach). A medial-to-lateral approach is carried out along the pancreatic body. The splenic flexure is then retracted medially by the assistant, and the mobilization is completed from the inferior splenic pole to the previous plane along the white line of Toldt. Coloparietal and colo epiploic detachments are performed, and splenic flexure takedown is thus completed. R1 is switched to the additional 8 mm trocar in the left flank. 

TME is carried out according to Heald’s embryologically based principles along the avascular plane to preserve the hypogastric nerve and the sacral venous plexus. The dissection starts posteriorly along the plane between the endopelvic visceral and parietal fascia. R1 is used to provide upward traction on the mesorectum, with the wrist joint in an L-shape to allow for a larger area of retraction; the assistant maintains cranial traction on the sigmoid colon, and R2 and R4 are the operative arms, with a bipolar grasper and a monopolar hook. The right lateral and the anterior plane are then dissected up to the seminal vesicles in a counterclockwise fashion; R1 is now used to lift the peritoneum of the Douglas pouch and vagina, and the seminal vesicles and prostate are freed and protected. The left lateral pelvic fascia is then dissected up to its lower portion until the pelvic nerve plexus is identified and the “bare rectum area” is visualized. 

The mesorectal dissection is performed in a cylindric fashion until past the level of the lesion and access to the levator ani plane is gained. During this phase in the lower mesorectum, a 0° camera may be helpful to achieve better visualization. Rectal transection is performed with a robotic stapler (60 mm or 45 mm according to the patient’s body habitus and pelvimetry) after the evaluation of the vascular perfusion of the rectal stump through the integrated fluorescence imaging system. Stapled end-to-end low/ultralow colorectal anastomosis or manual coloanal anastomosis are performed according to the tumor distance from the anal verge. A robotic top-down transabdominal intersphincteric resection (ISR) or targeted transection of the levator ani plane may be performed if a colonel anastomosis or abdominoperineal/extra levator abdominoperineal excision are required, respectively.

## 3. Laparoscopic versus Open Surgery: Long-Term Oncological Outcomes

Several RCTs have focused on the oncological adequacy of laparoscopic TME. Long-term results from the United Kingdom Medical Research Council Conventional versus Laparoscopic-Assisted Surgery in Colorectal Cancer (MRC-CLASICC), the Colorectal Cancer Laparoscopic or Open Resection (COLOR II), and the Comparison of Open versus Laparoscopic Surgery for mid or low Rectal Cancer After Neoadjuvant Chemoradiotherapy (COREAN) trials supported the use of laparoscopic surgery for colorectal cancer with no differences in overall survival, disease-free survival, and local recurrence rates when compared to open surgery.

Nevertheless, two recent RCTs have raised concerns about the oncological safety of laparoscopic TME. Pending clinical oncologic outcomes, the ALACART and ACOSOG studies failed to meet the criterion for non-inferiority for pathologic outcomes by using a combined metric including CRM, distal resection margin (DRM), and completeness of mesorectal excision. These two trials recently presented 2-year follow-up results as a secondary endpoint. The ACO-SOG showed no differences between the two groups. In contrast, the ALACART still questioned the oncological safety of the laparoscopic resections in the mid-term, even though no differences were found in the disease-free survival (DFS) and local recurrence rate (LLR) [14,15]. Moreover, the ALACART eligibility criteria for surgeons in the laparoscopic arm required just 30 rectal dissections, a relatively minor figure for a non-inferiority trial that may have affected the outcomes. 

MRC-CLASICC, COLOR II, and COREAN trials have reported 3-year DFS and OS ranging from 49.8% to 79.2% for the laparoscopic technique and 65.2% to 91.7% for the open surgery. Local recurrence rates ranged from 2.6% to 9.7%. The best oncological figures have been obtained in the COREAN trial, but, notably, the studied patient cohort was of Korean descent and, thus, may have presumably different characteristics compared to Western patients included in the other studies. Moreover, only three hospitals and surgeons with extensive laparoscopic experience enrolled patients, though only advanced extraperitoneal rectal cancers submitted to neo-adjuvant therapy were included. Therefore, results from COLOR II and MRC-CLASICC trials are more likely to represent the ‘real-life’ situation at that time (3-year DFS: 49.8% and 74.8%; 3-year OS: 65.2% and 86.7%; 3-year local recurrence rate: 5% and 9.7%, respectively). Several observational studies have investigated the oncological efficacy of the laparoscopic technique for rectal cancer. 

A German registry-based cohort compared more than 1500 rectal cancer resections from 2004 to 2013 and found that overall survival and cancer-specific survival was higher in the laparoscopic group [16]. A Spanish registry-based propensity score match study reported better overall and cancer-specific survival with lower local recurrences in the laparoscopic group [17]. A recent Swedish nationwide study also compared more than 8000 rectal cancer patients operated on from 2010 to 2016, reporting the non-inferiority of the laparoscopic approach with better 5-year overall survival [18]. The benefit of the minimally invasive approach (MIS) (laparoscopic and robotic) for the treatment of rectal cancer has been underlined in an American nationwide study [19]. They analyzed approximately 32,000 patients who underwent anterior resection for II–III stage rectal adenocarcinoma, reporting significantly improved short-term (length of hospitalization and 90-day mortality), pathological (CRM+ and number of harvested lymph nodes), and survival (1 and 5-year OS) outcomes for the MIS compared to the open arm.

## 4. Robotic versus Laparoscopic Surgery: Short-Term Outcomes and Pathological Outcomes

Laparoscopic TME is a challenging procedure, has a steep learning curve, and its penetration remains low after more than 20 years since the first cases were reported [20]. It maintains a high conversion rate to open surgery except for some studies from eastern countries, reaching a peak of 34% in the conversion rate as reported in the earliest published experiences (MRC-CLASICC). Though conversion rates reduced over time, maybe because of a learning effect, these figures seem to have plateaued, and a consistent value of 10–15% is still reported. Of more concern was the finding in the MRC-CLASICC trial that conversion was associated with a poorer long-term oncologic outcome irrespective of surgeon experience. In contrast, the meager conversion rate reported in the COREAN trial (1%) represents an exceptional case, maybe related to a rare and non-reproducible combination of patients’ characteristics and surgeons’ skillsets. A recent Norwegian national cohort study reported higher rates of positive CRM following converted laparoscopic TME, suggesting that non-optimal operative conditions (obese patients, narrow pelvis, or advanced tumors) requiring conversion to open surgery may affect the specimen quality resulting in inferior pathological outcomes [21].

Several meta-analyses have compared robotic versus laparoscopic TME, concluding that R-TME was associated with a lower conversion rate [22,23,24,25,26,27,28]. The preliminary results of the ROLARR trial have also shown a reduction in conversion rate for the most complex cases (high BMI, male sex, and low tumors) in the robotic group compared to its laparoscopic counterpart. Though the figures failed to meet the criteria of statistical significance, it is worthwhile to underline that all participating surgeons provided high-quality surgery in both groups, having performed a higher number of laparoscopic rectal resections compared to robotic operations (median number 91.5 vs. 50) before enrollment in this study. Moreover, as a superiority trial, a relative reduction of at least 50% in conversion rate was believed to be clinically relevant, and a value of 25% for laparoscopic surgery was considered realistic when the trial was designed. Unfortunately, such a high value no longer represents the actual situation, and the laparoscopic conversion rate turned out to be much lower (12.2%). A sample size of 471 patients could have been underpowered to meet the criteria for statistical significance.

Furthermore, Corrigan et al. conducted a multi-level logistic regression analysis adjusted for the operating surgeon experience to estimate a more realistic odds ratio for the conversion rate among the ROLLAR patients [29]. They concluded that the learning effect affected the primary outcome, and that robotic surgery offers a clear benefit in the conversion rate when performed by operators with proven caseload and experience. A recent retrospective paper by Rouanet et al. also showed a decreased conversion rate when performing robotic vs. laparoscopic anterior resection [30].

Retrospective data have shown a more rapid recovery and lower wound infections with the robotic approach compared to laparoscopy, reporting no other significant differences in the postoperative complication rate among these techniques [22,23,24,25,26,27,28,31,32,33,34,35]. The ROLARR trial reported similar outcomes with a leakage rate of 9.9% for the robotic group, slightly lower than laparoscopy with no statistical significance [12]. Previously reported complication (grade II or higher) rates in robotic-assisted surgery were 7.6% to 14.7%, and anastomotic leak rates were 4.5% to 10.5% [36,37,38,39]. The ALACART and the ACOSOG trials reported grade III–IV complication rates of 19% and 21.7% in the laparoscopic group, respectively. Though 2.1% and 7% leak rates have been reported in the same studies, values up to 10% and 12% have been found in the MRC-CLASICC and COLOR II trials. Rapid recovery with low complication rates may be necessary for these patients to avoid unnecessary delays in adjuvant therapy when indicated.

The comparison of the pathological outcomes shows how robotic surgery provides high-quality mesorectal resection with a comparable rate of CRM negativity and mean DRM [12,13,24,25,26,27,28,40,41,42,43,44]. Involvement of the circumferential resection margin is a well-established cause of local recurrence and worse long-term survival [45]. The ROL-LAR trial reported as a secondary endpoint of the study a CRM + of 5.1% for the robotic arm and 6.3% for the laparoscopic group with no statistical significance [12].

Regarding the pathological specimen quality, they reported a complete mesolectal excision of 76.4% and 77.6% for the two groups. The REAL trial, instead, reported a significant major rate of CRM positivity for the laparoscopic group (7.2% vs. 4.0%, *p* = 0.02), strengthened by a higher rate of complete mesorectal excision in the robotic arm (95.4% vs. 91.8%, *p* = 0.04) [13].

A recent meta-analysis has reported a better quality of the TME specimen following robotic TME [46]. However, it must be reported that the low quality and high heterogeneity of the studies therein are considered. 

In a recent propensity-adjusted American National Cancer Database analysis, Hopkins et al. found no differences in the negative CRM rate for approximately 8000 patients who underwent laparoscopic or robotic proctectomy after neo-adjuvant treatment [47]. Initial reports suggest excellent DRM and CRM negativity rates even in low tumors requiring multivisceral resections beyond the TME plane [48,49]. The benefits of robotic technology could thus be potentially maximized in the subset of patients affected by low and advanced rectal cancer, and, in this area, there is probably higher room for improvement.

## 5. Robotic Surgery: Long-Term Oncological Outcomes

Despite data in the literature showing how robotic TME is a feasible and secure technique providing promising clinical and pathological outcomes, only some studies are still available on mid- and long-term oncological results. 

Sammour et al. reported a 2.4% local recurrence rate analyzing 3-year data of 86 robotic TMEs, whereas Spanheimer et al. reported no local recurrences in 71 patients after 21 months of follow-up [42,43]. These results align well with the mid-term data from the ACOSOG and ALACART trials [14,15].

Aliyev et al. reported their 7-year follow-up data on a cohort of 115 patients with low rectal cancer (55 laparoscopic and 60 robotic). Median follow-up and oncological outcomes did not differ among the groups. Three-, five-, and seven-year OS were 88.6%, 80.4%, and 73.4% for the laparoscopic group and 90.4%, 86.3%, and 76.9% for the robotic counterpart. Three-, five-, and seven-year DFS rates for laparoscopy and robotics were 80.5%, 75.2%, and 70.4% vs. 84.4%, 81.4%, and 79.8%, respectively. Additionally, the local recurrence-free survival was almost comparable between the groups [50]. Lim et al. reported similar results by analyzing five years of follow-up data of 138 patients who underwent TME after neo-adjuvant therapy (74 robotic and 64 laparoscopic) [51]. Park et al. showed similar DFS and OS at five years for 236 matched patients (118 for each group) after laparoscopic or robotic TME. However, they reported a significantly lower distant recurrence rate in the robotic arm for patients with T3/T4 tumors (44.8% vs. 9.8), arguing a potential benefit of robotic resections for this subset of patients [52]. Huang et al., in multi-institutional retrospective work, documented a 5-year overall and disease-free survival of 91.1% and 86.3% for 605 patients with stages I–III rectal cancer who underwent robotic TME [53].

Table 1 summarizes the long-term oncological outcome of the studies mentioned above (open, laparoscopic, and robotic).

## 6. Conclusions

Though long-term survival data are sparse in the literature with no clear evidence that robotic surgery offers significant benefits in oncological outcomes over laparoscopy, robotic TME proved, in our experience, to be effective in treating extraperitoneal rectal cancer and provided high-quality surgery with good oncological outcomes. Long-term results of randomized trials are needed for a definitive conclusion with level I evidence. Prospective multi-centre studies focused on technically challenging low anterior resections are awaited.

## Figures and Tables

**Table 1:** Comparison of long-term oncological outcomes following R- (robotic), L- (laparoscopic), and O- (open) TME for rectal cancer—OS (overall survival); DFS (disease free survival); LRR (local recurrence rate); LRFS (local recurrence free survival).

	R-OS	L-OS	O-OS	R-DFS	L-DFS	O-DFS	R-LRR	L-LRR	O-LRR	R-LRFS	L-LRFS
**COLOR II [5,7]**	/	86.7% (3y)	83.6% (3y)	/	74.8% (3y)	70.8% (3y)	/	5% (3y)	5% (3y)	/	/
**COREAN [6]**	/	76.8% (10y)	74.1% (10y)	/	64.3% (10y)	59.3% (10y)	/	3.4% (10y)	8.9% (10y)	/	/
**ALACART [14]**	/	94% (2y)	93% (2y)	/	80% (2y)	82% (2y)	/	5.4% (2y)	3.1% (2y)	/	/
**ACOSOG [15]**	/	/	/	/	79.5% (2y)	83.2% (2y)	/	4.6% (2y)	4.5% (2y)	/	/
**Aliyev et al. [50]**	90.4% (3y)– 86.3% (5y)– 76.9% (7y)	88.6% (3y)–80.4% (5y)–73.4% (7y)	/	84% (3y)–81% (5y)–80% (7y)	80% (3y)–75% (5y)– 70% (7y)	/	/	/	/	96% (3y)–94% (5y)–90% (7y)	96% (3y)–92% (5y)–88% (7y)
**Huang et al. [53]**	91.1% (5y)	/	/	86.3% (5y)	/	/	3.0%	/	/	/	/
**Park er al. [52]**	92.8% (5y)	/	/	81.9% (5y)	/	/	2.3% (5y)	1.2% (5y)	/	/	/
**Lim et al. [51]**	90% (5y)	93.3% (5y)	/	76.8% (5y)	76% (5y)	/	2.7% (5y)	6.3% (5y)	/	/	

## Data Availability

Data can be found in the cited articles.
